# Influence of Genetic Ancestry on INDEL Markers of *NFKβ1*, *CASP8*, *PAR1*, *IL4* and *CYP19A1* Genes in Leprosy Patients

**DOI:** 10.1371/journal.pntd.0004050

**Published:** 2015-09-14

**Authors:** Pablo Pinto, Claudio Salgado, Ney Pereira Carneiro Santos, Sidney Santos, Ândrea Ribeiro-dos-Santos

**Affiliations:** 1 Laboratório de Genética Humana e Médica, Instituto de Ciências Biológicas, Universidade Federal do Pará, Belém, Pará, Brasil; 2 Núcleo de Pesquisas em Oncologia - NPO, Universidade Federal do Pará, Belém, Pará, Brasil; 3 Laboratório de Dermatoimunologia, Instituto de Ciências Biológicas, Universidade Federal do Pará, Belém, Pará, Brasil; Fondation Raoul Follereau, FRANCE

## Abstract

**Background:**

Leprosy is an insidious infectious disease caused by the obligate intracellular bacteria *Mycobacterium leprae*, and host genetic factors can modulate the immune response and generate distinct categories of leprosy susceptibility that are also influenced by genetic ancestry.

**Methodology/Principal Findings:**

We investigated the possible effects of *CYP19A1* [rs11575899], *NFKβ1* [rs28362491], *IL1α* [rs3783553], *CASP8* [rs3834129], *UGT1A1* [rs8175347], *PAR1* [rs11267092], *CYP2E1* [INDEL 96pb] and *IL4* [rs79071878] genes in a group of 141 leprosy patients and 180 healthy individuals. The INDELs were typed by PCR Multiplex in ABI PRISM 3130 and analyzed with GeneMapper ID v3.2. The *NFKβ1*, *CASP8*, *PAR1* and *IL4* INDELs were associated with leprosy susceptibility, while *NFKβ1*, *CASP8*, *PAR1* and *CYP19A1* were associated with the MB (Multibacilary) clinical form of leprosy.

**Conclusions/Significance:**

*NFKβ1* [rs28362491], *CASP8* [rs3834129], *PAR1* [rs11267092] and *IL4* [rs79071878] genes are potential markers for susceptibility to leprosy development, while the INDELs in *NFKβ1*, *CASP8*, *PAR1* and *CYP19A1* (rs11575899) are potential markers for the severe clinical form MB. Moreover, all of these markers are influenced by genetic ancestry, and European contribution increases the risk to leprosy development, in other hand an increase in African contribution generates protection against leprosy.

## Author Summary

Leprosy is an infectious disease caused by *Mycobacterium leprae*, which can carry to skin lesions and affect peripheral nerves, which cause physical and motor injuries on the patients. Moreover, leprosy, may be classified in two major groups, based on clinical manifestations in Paucibacillary (PB) or Multibacillary (MB), and these phenotype may be influenced by host immune response; that can be controlled by genetics factors that can be useful like future panel of biomarkers to leprosy, and it’s related with the different genetic background of population studied. Therefore, we conducted a study to evaluate seven INDEL polymorphisms in seven genes involved in modulation of the host immune response, and consequently can modulated o phenotype showed through the disease, to identify possible susceptibility markers of leprosy. However this analysis can be spurious on presence of population structure, common in admixture population like the Brazilian, thus we evaluate like the influence of genetic ancestry can modulated the disease risk.

## Introduction

Leprosy is an insidious infectious disease caused by the obligate intracellular bacteria *Mycobacterium leprae* that affects the skin and peripheral nerves, causing a chronic granulomatous infection [1]. Leprosy patients may be classified in two major groups, based on clinical manifestations using a simple system introduced by the WHO (World Health Organization) in 1982. Paucibacillary (PB) is the primary characteristic of Tuberculoid (TT) leprosy and is characterized by a few lesions and scarce bacilli, and Multibacillary (MB) is the primary characteristic of anergic Lepromatous (LL) leprosy. From an epidemiological perspective, the situation in Brazil is critical because, along with India and Indonesia, it has the highest rate of new cases detected worldwide [2, 3, 4].

In addition to the system introduced by WHO in 1982, the use of histological and immunological criteria as described by Ridley-Jopling further improves definition of Borderline cases. According to this classification, TT (tuberculoid-tuberculoid) patients, who have the PB type, exhibit a strong cellular immune response (CIR) mediated by Th1, and a negative skin smear test. In contrast, LL (lepromatous-lepromatous) patients have a weak or absent CIR and a highly positive skin smear associated to an humoral immune response. In the middle of this spectrum are a large number of borderline patients, which together with LL comprise the MB pole, with symptoms varying from weak to strong CIR and negative to positive skin smears [5, 6].

The regulation of the host immune response and manifestation of disease clinical between types PB (better) and MB (severe) involves cytokine and others mediators produced by various subtypes of T cells. In PB, an inflammatory immune response is mediated by Th1 cells that express pro-inflammatory interleukins that stimulate macrophages and phagocytosis mechanisms to inhibit bacillary growth and kill mycobacteria [2,7–9]. On the other hand, MB patients have an intense Th2 immune response with production of anti-inflammatory cytokines in addition to the specific anti-PGL-1 (phenolic glycolipid 1) antibody. This mechanism does not block bacillary growth and contributes to the host’s inability to resist the development of severe disease [2,8,9–11].

Recent studies have investigated genetic markers, usually innate immune response genes, as possible susceptibility factors for leprosy because the SNPs in these genes can modulated the host immune response and consequently lower host resistance to bacillus growth [6,12,13]. However, few studies have investigated INDEL polymorphisms (insertion-deletion) in immune response genes in leprosy. Moreover, such polymorphisms present interesting features as genetic markers because i) INDELs are spread throughout the human genome, ii) INDELs derive from a single event (they do not present homoplasy), iii) small INDELs can be analyzed using short amplicons, which improves amplification of degraded DNA and facilitates multiplexing reaction, iv) INDELs can create abrupt changes in the normal function of the gene and v) INDELs can be easily genotyped using a simple dye-labeling electrophoretic approach [14]. The current study select eight INDEL in seven genes (*CYP19A1*, *NFKβ1*, *IL1α*, *CASP8*, *UGT1A1*, *PAR1*, *CYP2E1*, and *IL4*), which have relation with the immune response modulation in leprosy patients, beside literature that demonstrate these molecular markers like functional polymorphisms that alter transcriptional activity of the gene, and consequently the immunological phenotype against the bacilli. Additionally these INDELs can be able to contribution to construction a possible panel of susceptibility markers.

However, from the genetic point of view, Brazil is recognized as having one of the most heterogeneous populations in the world, with important genetic information being contributed by three main continental groups, Europeans, Africans and Amerindian, resulting in a genetically very diverse modern Brazilian population [15]. Therefore, analysis of genetic markers in complex diseases may result in spurious results due to population substructure [16], and it is important to perform the genomic ancestry control, especially in populations with a high degree of interethnic admixture [14].

The objective of this study was to investigate eight INDEL polymorphisms in seven genes involved in modulation of the host immune response, including *CYP19A1* [rs11575899], *NFKβ1* [rs28362491], *IL1α* [rs3783553], *CASP8* [rs3834129], *UGT1A1* [rs8175347], *PAR1* [rs11267092], and *CYP2E1* [INDEL 96pb], besides one VNTR (variable number tandem repeat) of 70 bp on intron 3 of *IL4* [rs79071878] in a group consisting of 141 leprosy patients and 180 healthy individuals, to identify possible susceptibility markers of leprosy and evaluate the influence of genetic ancestry on disease risk.

## Materials and Methods

### Ethics statement

The project was approved by the Pará Federal University ethics committee (N° 197/07).

### Samples

We investigated 141 leprosy patients who attended the Dr Marcello Candia Reference Unit in Sanitary Dermatology of the State of Pará (UREMC), in Marituba, Pará, Brazil between January 2008 and December 2009. All patients were informed about the study before they signed informed consent forms. Since 2002, UREMC registered between 308 and 472 leprosy patients (mean: 408 cases per year). Of the 765 leprosy cases registered in 2008 and 2009 alone, 141 (18.43%) were randomly selected for this study. These patients were divided according to Ridley-Jopling classification [5] into Paucibacillary (TT: PB 31) and Multibacillary (BT, BB, BL and LL: MB 110) groups. A total of 180 healthy individuals who were unrelated, without leprosy or other chronic diseases and from the same geographic area as each other were chosen for the control group. Leprosy patient’s descriptions were made previously [6]. These subjects were asked to participate in the study after being informed about the study objectives and signing informed consent forms.

### DNA extraction

DNA extraction was performed as previously described by phenol-chloroform method [6, 17]. The DNA concentration was determined by spectrophotometry (Themo Scientific NanoDrop 1000, NanoDrop Technologies, Wilmington, US).

#### Multiplex Typing

DNA samples were typed for the 7 biallelic INDELs and 1 VNTR of 70 bp. Each multiplex PCR was performed in a final volume of 10 μL containing 5 μL of QIAGEN Multiplex PCR Kit, 1 μL of Q-solution, 1 μL of primer mix (Forward + Reverse primers at a concentration of 2 μM each), 1 μL of DNA (10 ng) and 2 μL of water. The fluorescent molecules 6FAM and HEX were inserted at the 5’ position of each primer (forward or reverse). All PCR reactions were performed on the Thermocycle Veriti-96 Well Thermal Cycler (Life Technologies, CA, USA). Before capillary electrophoresis, 1.0 μL PCR product was added to 8.5 μL deionized formamide HI-DI (Life Technologies, CA, USA) and 0.5 μL GeneScan 500 LIZ size standard (Life Technologies, CA, USA). DNA fragments were separated using an ABI PRISM 3130 Genetic Analyzer (Life Technologies, CA, USA) and analyzed with GeneMapper ID v3.2 software (Life Technologies, CA, USA). This method is similar like described in a paper of INDEL markers of our group [14].

### Ancestry Informative Marker (AIM)

Individual interethnic admixture was estimated using a panel of 48 ancestry informative markers (AIMs) as previously described [6, 14].

### Statistical analysis

The allelic frequencies between healthy individuals and leprosy patients and between PB and MB patients were estimated by gene counting. Deviation from the Hardy-Weinberg equilibrium was assessed using chi-squared tests, using the Arlequin v3.5 software [18], and p-value of HWE was corrected by Bonferroni methods.

Differences between leprosy patients and healthy individuals and between PB and MB patients with respect to age, gender and genetic ancestry were estimated using Student’s t-Test, Fisher’s exact test and Mann-Whitney tests, respectively. The association of markers between groups was analyzed by logistic regression tests, all the test were corrected by FDR (False Discovery Rate) method, and all tests were performed using the statistical package under R calculation. A two-tailed p-value < 0.05 was considered statistically significant.

The individual contributions of European, African and Amerindian genetic ancestry were estimated using the STRUCTURE 2.3.3 program assuming three parental populations (European, African and Amerindian), a burn-in period of 200,000, and 200,000 Markov Chain Monte Carlo repetitions after burn-in [16]. The differences in allelic frequencies between leprosy cases and the healthy individuals for markers analyzed following an adjustment for population stratification was performed using the STRAT software program with 10,000 simulations [16].

## Results

The data of clinical and demographic distribution of leprosy patients and healthy individuals is shown in Table 1. The mean age was higher in healthy individuals (55.7±12 versus 43.3±21, p<0.001), and male patients were more frequent among leprosy patients (97 [68.8%] versus 65 [36.1%], p<0.001). Analysis of ethnicity showed that the mean frequency of Africans was higher among leprosy patients (0.284 versus 0.236, p<0.001) and Europeans were more frequent in healthy individuals (0.461 versus 0.427, p = 0.004).

**Table 1:** Demographic and clinical characteristic of the sample of leprosy patients and healthy individuals.

Variables	LEPROSY PATIENTS (n = 141)	HEALTHY INDIVIDUALS (n = 180)	*p value* (IC-95%)
Age^a^	43.3±21	55.7±12	<0.001
Gender^b^ (M/F)	97(68.8%)/44(31.2%)	65(36.1%)/115(63.9%)	<0.001
**Genetic Ancestry** ^**c**^			
African	0.284±0.11	0.236±0.04	<0.001
European	0.427±0.13	0.461±0.06	0.004
Ameridian	0.289±0.11	0.303±0.09	0.094

^a^ *t*-Test of Student;

^b^Fisher's Exact Test;

^c^ Mann-whitney test; The data are show like mean ± standard deviation.

The frequencies of INDELs for the eight (8) genes analyzed in leprosy patients and healthy individuals are show in Table 2. For the polymorphism in *IL4* (VNTR of 70 bp), only two alleles were identified in the sample. One allele had two repeats of 70 bp (allele A1) and the other had three repeats of 70 bp (allele A2), suggesting theses alleles are biallelic markers. All the polymorphisms analyzed were according to the Hardy Weinberg equilibrium, therefore the association analysis were performed with regression logistic test and differences in allelic frequencies were corrected by frequencies of ancestry markers informative.

**Table 2:** Allele frequencies of INDELs for the eight investigated genes.

GENE	LEPROSY PATIENTS (n = 141)	HEALTHY INDIVIDUALS (n = 180)
*CYP19A1* (rs11575899)		
INS	0.574	0.558
DEL	0.425	0.442
HWE	0.998	0.998
*NFKβ1* (rs28362491)		
INS	0.546	0.467
DEL	0.454	0.533
HWE	0.281	0.266
*IL1α* (rs3783553)		
INS	0.617	0.544
DEL	0.383	0.456
HWE	0.898	0.779
*CASP8* (rs3834129)		
INS	0.557	0.586
DEL	0.443	0.414
HWE	0.215	0.996
*UGT1A1* (rs8175347)		
INS	0.411	0.325
DEL	0.589	0.675
HWE	0.280	0.487
*IL4* (rs79071878)		
A_2_ ^a^	0.660	0.581
A_1_ ^b^	0.340	0.419
HWE	0.521	0.876
*PAR1* (rs11267092)		
INS	0.320	0.217
DEL	0.680	0.783
HWE	0.06	0.196
*CYP2E1*		
INS	0.088	0.083
DEL	0.911	0.916
HWE	0.600	0.994

HWE = p-value for Hardy Weinberg equilibrium after Bonferroni correction;

^a^ Allele with three repeats of 70 pb;

^b^ Allele with two repeats of 70 pb

When the INDELs were analyzed by logistic regression, the genes *NFKβ1* and *PAR1* showed statistically significant differences associated with the presence of the DEL allele (p = 0.016 and p = 0.022, respectively) and both were associated like protection factors to not developing the disease (OR[IC95%] = 0.50[0.27–0.88] and OR[IC95%] = 0.35[0.14–0.86], respectively), for these genes was found a dominance effect DEL allele, that increase your protection capacity in general population. The *CASP8* showed significant differences associated with the presence of the DEL/DEL homozygous genotype and was associated with a risk factor for leprosy development (p = 0.017; OR[IC95%] = 2.33[1.16–4.69]) (Table 3). The analysis of allele frequency differences was then corrected for the influence of genetic ancestry on population structure, and the results showed that the DEL allele of *PAR1* gene and the allele A_1_ of *IL4* is more frequent in healthy individuals (p = 0.018 and p = 0.019, respectively) (Table 3), these results shown the importance of statistical correction in admixture population, in order to exhibit differences covert by structure population.

**Table 3:** Allelic and genotypic distribution between leprosy patients and healthy individuals to markers associated whit susceptibility to leprosy.

GENE	LEPROSY PATIENTS n(%)	HEALTHY INDIVIDUALS n(%)	*p* ^a^	OR (IC95%)^b^	*p*_*STRAT*_ ^c^
*NFKβ1* (rs28362491)					
INS/INS	45(31.9%)	35(19.4%)		1	
INS/DEL	64(45.4%)	98(54.4%)			
DEL/DEL	32(22.7%)	47(26.1%)	0.559	0.83(0.64–2.34)	
[DEL]carriers	96(68.1%)	145(80.5%)	**0.016**	**0.50(0.27–0.88)**	
INS	0.546	0.467			
DEL	0.454	0.533			0.243
*PAR1* (rs11267092)					
INS/INS	19(13.5%)	11(6.1%)		1	
INS/DEL	52(36.9%)	56(31.1%)			
DEL/DEL	70(49.6%)	113(62.8%)	0.094	0.64(0.39–0.86)	
[DEL]carriers	122(86.5%)	169(93.9%)	**0.022**	**0.35(0.14–0.86)**	
INS	0.320	0.217			
DEL	0.680	0.783			**0.018**
*CASP8* (rs3834129)					
INS/INS	50(35.5%)	62(34.4%)		1	
INS/DEL	57(40.4%)	87(48.3%)			
DEL/DEL	34(24.1%)	31(17.2%)	**0.017**	**2.33(1.16–4.69)**	
[DEL]carriers	91(64.5%)	118(65.5%)	0.413	0.80(0.47–1.36)	
INS	0.557	0.586			
DEL	0.443	0.414			0.123
*IL4*(rs79071878)					
A_2_/ A_2_	63(44.7%)	60(33.3%)		1	
A_2_/ A_1_	60(42.6%)	89(49.4%)			
A_1_/ A_1_	18(12.8%)	31(17.2%)	0.132	0.56(0.26–1.18)	
[A_1_]carriers	78(55.4%)	120(66.6%)	0.088	0.63(0.37–0.84)	
A_2_ ^d^	0.660	0.581			
A_1_ ^d^	0.340	0.419			**0.019**

^**a**^ *p-value* obtained for logistic regression adjusted by age, gender and genetic ancestry;

^b^ Adjusted Odds Ratio (OR);

^c^ p-value after correction for population structure;

^d^ A_1_—allele with two tandem repeats A_2_—allele with three tandem repeats.

Table 4 summarizes the clinical and demographic characteristics of leprosy patients grouped according to clinical manifestation in PB (Paucibacillary) and MB (Multibacillary) groups, and the only significant difference was observed for age (p = 0.003), with a higher mean age in MB patients (45.7±22 versus 34.9±15). When the INDELs were analyzed by logistic regression, *NFKβ1* showed significant differences like risk factor associated with the presence of the allele DEL in MB patients (p = 0.024; OR[IC95%] = 2.64[1.13–6.19]), of contradictory way the dominance effect of DEL allele seem protect against the development of leprosy, but when the disease is established your effect seem inefficient to combat to bacilli. *PAR1* showed significant differences associated with the presence of homozygous DEL/DEL genotype in PB patients (p = 0.031; OR[IC95%] = 0.41[0.17–0.96]) (Table 5). The analysis of allele frequency differences were corrected for population structure and showed that the DEL allele of *CASP8* is more frequent in PB patients (p = 0.003), while the DEL allele of *CYP19A1* is more frequent in MB patients (p = 0.007) (Table 5).

**Table 4:** Demographic and clinical characteristics of the sample according with clinical form of leprosy.

Variables	LEPROSY PATIENTS		*p value* (IC-95%)
	PB (n = 31)	MB(n = 110)	
Age^a^	34.9±15	45.7±22	**0.003**
Gender^b^ (M/F)	19(63.3%)/11(36.7%)	78(70.9%)/32(29.1%)	0.504
**Genetic Ancestry** ^**c**^			
Afric	0.290±0.10	0.282±0.12	0.534
European	0.419±0.09	0.429±0.13	0.964
Ameridian	0.289±0.10	0.288±0.11	0.648

^a^ *t*-Test of Student;

^b^Fisher's Exact Test;

^c^ Mann-whitney test; The data are show like mean ± standard deviation.

**Table 5:** Allelic and genotypic distribution between leprosy patients grouped according clinical form PB or MB.

GENE	PATIENTS PB n(%)	PATIENTS MB n(%)	*p* ^a^	OR (IC95%)^b^	*p*_*STRAT*_ ^c^
*NFKβ1* (rs28362491)					
INS/INS	15(48.4%)	30(27.3%)		1	
INS/DEL	13(41.9%)	51(46.4%)			
DEL/DEL	3(9.7%)	30(27.3%)	0.119	2.78(0.76–10.07)	
[DEL]carriers	16(51.6%)	81(73.7%)	**0.024**	**2.64(1.13–6.19)**	
INS	0.694	0.495			
DEL	0.306	0.504			0.410
*PAR1* (rs11267092)					
INS/INS	5(16.1%)	14(12.7%)		1	
INS/DEL	5(16.1%)	47(42.7%)			
DEL/DEL	21(67.7%)	49(44.5%)	**0.031**	**0.41(0.17–0.96)**	
[DEL]carriers	26(83.9%)	96(87.2%)	0.259	1.98(0.60–6.55)	
INS	0.241	0.340			
DEL	0.759	0.660			0.223
*CASP8* (rs3834129)					
INS/INS	7(22.6%)	43(39.1%)		1	
INS/DEL	16(51.6%)	41(37.3%)			
DEL/DEL	8(25.8%)	26(23.6%)	0.579	0.76(0.29–1.97)	
[DEL]carriers	24(77.4%)	67(60.9%)	0.114	0.46(0.18–0.90)	
INS	0.484	0.577			
DEL	0.516	0.423			**0.003**
*CYP19A1*(rs11575899)					
INS/INS	14(45.2%)	32(29.1%)		1	
INS/DEL	17(54.8%)	53(48.2%)			
DEL/DEL	-	25(22.7%)	0.998	-	
[DEL]carriers	17(54.8%)	78(70.9%)	0.082	2.11(1.00–4.93)	
INS	0.726	0.532			
DEL	0.274	0.468			**0.007**

^**a**^ *p-value* obtained for logistic regression adjusted by age, gender and genetic ancestry;

^b^ Adjusted Odds Ratio (OR);

^c^ p-value after correction for population structure.

**Fig 1:**
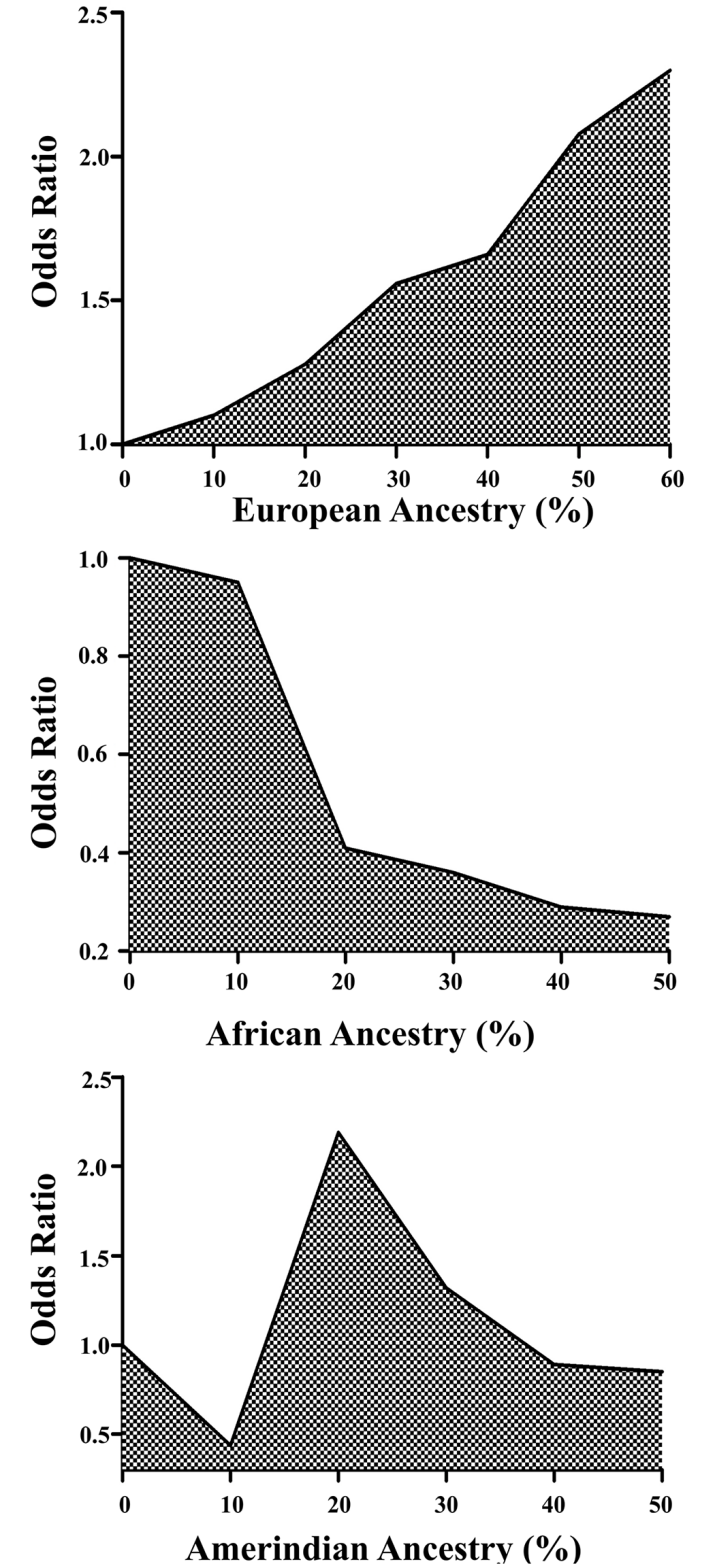
In a comparison of 141 leprosy patients and 180 healthy individuals, events with statistically significant (p<0.05) differences can be categorized into six categories of individuals with African and European genetic ancestry (10%>20%> 30%>40%>50%>60%), the p-values were adjusted by age and gender. The analysis in individuals with Amerindian ancestry was not statistically significant.

Fig 1 shows the OR (odds ratio) values obtained from leprosy patients and healthy individuals within groups having distinct level of ancestry composition. The figure shows that greater frequency of European ethnic between the groups (leprosy patients and healthy individuals), higher is the risk for developing leprosy, while the smaller the frequency of the African ethnic, lower is the risk for developing leprosy. No statistically significant values were obtained for the analysis of the Amerindian group.

These results are better understood on frequencies distribution, according with range of ancestry contribution (S1 Table). For African ancestry 99.4% of health individual is closed between 0% and 50% of African contribution (range that have p<0.05 on Fig 1), moreover the contribution range of 10% to 30% is closed 81.7% of health individual, in this range the Fig 1 have more decline of OR value, that showed the higher protection effect of African ancestry. To European ancestry, 61.7% of leprosy patients is closed between 40% to 80% of European contribution, while 87.2% of health individuals is closed between 0% to 50% of European contribution. Additionally, for the contribution range between 60% to 80% we observed 17% of all patients, while no healthy individual was observed this range, these data show that leprosy patients have higher European contribution compared with healthy individuals. Take together the Fig 1 and S1 Table shown that to leprosy patients of an admixture population, like Brazil, African ethnic generates protection against the development of disease, and the opposite is also truth for European ethnic.

## Discussion

NF-κB belongs to family of protein transcription factors that modulate many inflammatory processes. In the resting state, IκBα (inhibitor of NF-kβ activity) sequesters NF-κB in the cytoplasm and prevents its activity, but in response to specific stimuli, IkBα is ubiquitinated and degraded allowing NF-kB to migrate to the nucleus and stimulate the transcription of proinflammatory genes [19,20]. The allele DEL (rs28362491) has been shown to be associated with a decrease of transcriptional activity of variety genes of immune response [21] and with auto immune disease such as Systemic Sclerosis [22] and lupus erythematosus [23].

The role of NF-kβ in leprosy is not clear, and studies linked to expression of NF-kβ have suggested that lower expression is common in leprosy patients [24,25]. Our results suggest that the DEL carries genotype induces protection against leprosy (Table 3), although a comparison of PB and MB patients also suggests that DEL behaves like a risk factor for the development of the severe clinical form of MB (Table 5). Because the transcription of NF-kβ is mediated by specific stimuli, such as the presence of *M*. *leprae* [24], it is conceivable that the presence of DEL confers risk to MB leprosy.

*PAR1* is a receptor of the PAR family of proteins that belong to a unique group of G protein—coupled receptors. In particular, *PAR1* protein is present in a variety of cells like platelets, endothelia, epithelial, neurons, fibroblasts, smooth muscle, leukocytes and tumor lines [26]. This receptor has been shown to be involved in many natural physiological processes, that involve inflammation like the systems cardiovascular, respiratory and central nervous and in embryogenesis, cancer and inflammation [27]. *PAR1* suppresses T helper type 1 (Th1) and T helper type 17 (Th17) cells and the secretion of IL-12 and IL-23, thereby resulting in the inhibition of pro-inflammatory responses [28]. The allele of insertion (INS) of INDEL studied (rs11267092) has been shown to increase gene transcription [29] and therefore, it is a risk allele for leprosy. Our results suggest that the presence of DEL induces protection against leprosy (Table 3), and the DEL/DEL genotype confers protection against the development of clinical forms of MB (Table 5), thus this genotype of *PAR1* gene can suppresses cellular infiltration and increase both Th1 and Th17 responses to infection. Moreover, analyses of macrophages revealed that secretion of IL-12 and IL-13, two cytokine that play role key on cellular immunity Th1 and Th17, can be suppressed by *PAR1* activation. Furthermore, *PAR1* can suppress interferon regulatory factor 5 (IRF5), that play role key like transcription factor for IL-12 and IL-23, which modulates the sub sets of cellular immunity. Thereby the suppression of IRF5 and IL-12/23 secretion by *PAR1* gene, can provides a novel mechanism by which the host suppresses the Th1 and Th17response to infection, and dysregulation of this process can likely an important factor in the susceptibility of some individuals to leprosy [28].

Macrophages with a high load of *M*. *leprae* have been shown to undergo apoptosis, and this mechanism is under the control of cytokines [30]. In leprosy patients, the immune system is overburdened with bacilli, and most likely the continuous activation of T cells by circulating *M*. *leprae* antigens leads to apoptosis and to a reduction of peripheral lymphocytes and other immune effector cells in these patients with the regulation of apoptosis involved in the stimulation and activation of caspase-8 [31]. The allele DEL (rs3834129) cause a decrease in *CASP8* transcription and a reduction in apoptosis [32], thereby improving the bacillary load. Our results suggested that the DEL/DEL genotype (Table 3) and the high frequency of DEL allele (Table 5) can raise the bacillary load and thus confers a risk to leprosy development.

Interleukin-4 (IL-4) is a key cytokine secreted by Th2 lymphocytes, eosinophils and mast cells that induces the activation and differentiation of B cells and the development of the Th2 subset of lymphocytes, which is ineffective in combating leprosy [33]. Our analysis of the VNTR on intron 3 of the *IL4* gene (rs79071878) revealed two common alleles with two (A_1_) and three (A_2_) tandem repeats. Of these, A_2_ allele is known to be a high producer of IL-4 [34]. Our results indicate that allele A_2_ is more frequent in leprosy patients compared to healthy individuals, consistent with the fact that higher levels of *IL4* would be ineffective in controlling the growth of bacilli (Table 3).

The conversion of androgens to estrogens, catalyzed by aromatase encoded by the *CYP19A1* gene, is the primary pathway of estrogen production in humans [35]. The levels of these hormones are important in leprosy patients and it has been demonstrated that androgen levels are significantly lower in leprosy patients compared to healthy control subjects [36]. Moreover, there is an inverse correlation between plasma androgen levels and secretion of inflammatory cytokines, suggesting that high plasma androgen levels can be less effective in inhibiting bacillus growth [37]. The DEL allele (rs11575899) has previously been reported to have a negative effect on aromatase activity [38], and our results show that the DEL allele is more frequent in MB patients (Table 5). We hypothesize that the DEL allele can decrease aromatase activity and increase androgen levels, resulting in an overall reduction in effective combat of bacillary growth and development of the severe clinical form MB.

It is unclear whether leprosy originated in Asia or Africa. However, leprosy is believed to have been introduced into Europe from India, and the incidence was high in Europe during the Middle Ages until approximately 1870 when the number of cases dramatically reduced because of socioeconomic development [39,40,41]. It is believed that leprosy was introduced in Brazil primarily by the Spanish and Portuguese [41]. Estimates indicate that before the arrival of colonizers, approximately 2.5 million natives lived in Brazil, and during the European immigration in the first three centuries, approximately 500,000 individuals came from Portugal and approximately 3.5 million Africans were brought into Brazil through slave trade [14]. Therefore, there is evidence of a so-called directed admixture process involving predominantly European, Native American and African people [42–45].

Our data indicates that the contribution of different ethnic groups to the composition of the current Brazilian population can generate different rates of risk for leprosy development according to the level of inter-ethnic composition of the individuals involved. Our analysis suggests that an increase in European contribution increases the risk of leprosy development, while an increase in African contribution decreases the risk for leprosy development and the Amerindian contribution does not result in any statistically significant differences (Fig 1).

The introduction of leprosy in Brazil primarily can it be accredited to the slave trade, but no only for this reason. Slaves were firstly there from Africa, and in succeeding years the number these slaves were increased, but was not common between they the clinical manifestation of leprosy, because these slaves were from region of the Africa where leprosy was comparatively rare. Moreover isn't doubt that the Portuguese and, to a less degree, Dutch, French and Spaniards were responsible by introduction of leprosy in Brazil, on period of country colonization. Additionally, data showed that as early, as 1419, the disease was common in Portuguese and epidemiologically in this time the leprosy was very prevalent in Europe, and particularly in Portugal [41]. Therefore, our data of risk of leprosy according the different ethnic groups compositions is consistent with the higher numbers of settlers Portuguese that came to Brazilian that probably increases the frequencies of alleles of susceptibility on Brazilian population [14, 42–45]. In other hand, the African contribution may have increase the frequencies of allele that confer protection against to leprosy.

Comparative analyses of the four *M*. *leprae* genomes (India, Thailand, Brazil and US) have revealed little clonal differences. Thus, the patterns of global human migration routes, during the past 100,000 years, corroborate and suggest that leprosy probably originated in Africa [46]. African-descendants in admixture populations can be less susceptible to the leprosy bacilli, probably because of genetic polymorphisms accumulated during these times, in gene that can modulate the immune response on infection combat. Furthermore African humans are the more genetically diverse population in the world consequently, by selection bias, genetic polymorphisms accumulated that confer protection against disease, can be present in this population and your descendants.

Of point view epidemiological, the situation of African and Americas region is critical, and is associated the socioeconomic challenges related to the disease, but genetics components also are important to disease knowledge [4]. Thus understanding of like genetic ancestry, in admixture population, can to influence genetic susceptibility is essential to avoid spurious results. In conclusion, our study shows that the *NFKβ1* [rs28362491], *CASP8* [rs3834129], *PAR1* [rs11267092] and *IL4* [rs79071878] genes are possible markers for the susceptibility to development of leprosy and the severe clinical form MB. Moreover, after correcting for population structure within an admixture population, the results show that different levels of ethnic group composition can generate different OR rates for leprosy susceptibility.

## Supporting Information

S1 Table Distribution of percent range of African and European genetic ancestry between leprosy patients and healthy individuals. (DOCX)

## References

[1] Alcais A, Mira M, Casanova JI, Schurr E, Abel I. Genetic dissection of immunity in leprosy. Current Opinion in Immunology. 2005; 17: 44–48. 15653309 10.1016/j.coi.2004.11.006

[2] Elizabeth AM, William RB, James CV, Hawn TR. Leprosy and the Human Genome. Microbiol. Mol. Biol. 2010; 74(4):589–620. 10.1128/MMBR.00025-10 PMC3008172 21119019

[3] Salgado CG, Ferreira DVG, Frade MAC, Guimarães LS, Silva MB, Barreto JG. High Anti—Phenolic Glycolipid-I IgM Titers and Hidden Leprosy Cases, Amazon Region. Emerging Infectious Diseases. 2012; 19: 46–49. 10.3201/eid1805.111018 PMC3358075 22515845

[4] World Health Organization (WHO). Global leprosy: update on the 2012 situation. WHO N°35. 2013; 88: 365–380. Available: http://www.who.int/wer/2013/wer8835.pdf?ua=1 24040691

[5] Ridley DS, Jopling W H. Classification of leprosy according to immunity: a five-group system. Int. J. Leprosy. 1966; 34:255–73. 5950347

[6] Pinto P, Salgado CG, Santos N, Alencar DO, Santos S, et al Polymorphisms in the CYP2E1 and GSTM1 Genes as Possible Protection Factors for Leprosy Patients. PLoS ONE. 2012; 7(10): e47498 10.1371/journal.pone.0047498 23077626 PMC3471857

[7] Foss NT.Immunological aspects of Leprosy. Medicina Ribeirão Preto. 2007; 30: 335–339.

[8] Britton WJ, Lockwood DN. Leprosy Lancet. 2004; 363:1209–1219. 15081655 10.1016/S0140-6736(04)15952-7

[9] Scollard DM, Adams LB, Gillis TP, Krahenbuhl JL, Truman RW, Williams DL.The continuing challenges of leprosy. Clin. Microbiol. Ver. 2006; 19:338–381. 10.1128/CMR.19.2.338-381.2006 PMC1471987 16614253

[10] Salgame PM, Bloom YBR, Modlin RL. Evidence for functional subsets of CD4_ and CD8_ T cells in human disease: lymphokine patterns in leprosy. Chem. Immunol. 1992; 54:44–59. 1358110

[11] Yamamura M, Uyemura K, Deans RJ, Weinberg K, Rea TH, Bloom BR, et al Defining protective responses to pathogens: cytokine profiles in leprosy lesions. Science. 1992; 254:277–279. 10.1126/science.254.5029.277 1925582

[12] Garcia P, Alencar D, Pinto P, Santos N, Salgado C, Sortica VA, et al Haplotypes of the IL10 gene as potential protection factors in leprosy patients. Clin Vaccine Immunol. 2013; 10;20(10):1599–603. 10.1128/CVI.00334-13 23966553 PMC3807203

[13] Bobosha K, Wilson L, van Meijgaarden KE, Bekele Y, Zewdie M, et al T-Cell regulation in Lepromatous Leprosy. PLoS Negl Trop Dis. 2014; 8(4): e2773 10.1371/journal.pntd.0002773 24722473 PMC3983090

[14] Santos NPC, Ribeiro-Rodrigues EM, Ribeiro-dos-Santos AKC. Assessing individual interethnic admixture and population substructure using a 48 insertion-deletion ancestry informative markers panel. Hum Mutat. 2010; 31(2): 184–90. 10.1002/humu.21159 19953531

[15] Palha T, Gusmão L, Ribeiro-Rodrigues E, Guerreiro JF, Ribeiro-dos-Santos A, et al Disclosing the Genetic Structure of Brazil through Analysis of Male Lineages with Highly Discriminating Haplotypes. PLoS ONE. 2012; 7(7): e40007 10.1371/journal.pone.0040007 22808085 PMC3393733

[16] Pritchard JK, Stephens M, Donnelly P. Inference of population structure using multilocus genotype data.Genetics. 2000; 155:945–959. 10835412 10.1093/genetics/155.2.945 PMC1461096

[17] Sambrook J, Fritsch F, Maniatis T. Molecular Cloning: A Laboratory Manual. Cold SprHarb Laboratory 1989; NY 2nd edition.

[18] Excoffier L. Laval G., and Schneider S.. Arlequin ver. 3.0: An integrated software package for population genetics data analysis. Evolutionary Bioinformatics Online. 2005; 1:47–50. PMC2658868 19325852

[19] Ali S, Hirschfeld AF, Mayer ML, Fortuno ES, Corbett A, Kaplan M, et al Functional Genetic Variation in NFKBIA and Susceptibility to Childhood Asthma, Bronchiolitis, and Bronchopulmonary Dysplasia. J Immunol. 2013; 190: 3949–3958. 10.4049/jimmunol.1201015 23487427

[20] Andersen V, Christensen J, Ernst A, Jacobsen BA, Tjønneland TA, Krarup HB, et al Polymorphisms in NF-κB, PXR, LXR, PPARγ and risk of inflammatory bowel disease. World J Gastroenterol. 2011; 17(2): 197–206. 10.3748/wjg.v17.i2.197 21245992 PMC3020373

[21] Karban AS, Okazaki T, Panhuysen CI, Gallegos T, Potter JJ, Bailey-Wilson JE, et al Functional annotation of a novel NFKB1 promoter polymorphism that increases risk for ulcerative colitis. Hum Mol Genet. 2004; 13: 35–45. 14613970 10.1093/hmg/ddh008

[22] Salim PH, Jobim M, Bredemeier M, Chies JAB, Brenol JCT, Jobim LF, ET al Interleukin-10 Gene Promoter and NFKB1 Promoter Insertion/Deletion Polymorphisms in Systemic Sclerosis. Scandinavian J. Immunology. 2013; 77:162–168. 10.1111/sji.12020 23237063

[23] Cen H, Zhoua M, Lenga RX, Wanga W, Fenga C, Li B et al Genetic interaction between genes involved in NF-KB signalingpathway in systemic lupus erythematosus. Molecular Immunology. 2013; 56:643–648. 10.1016/j.molimm.2013.07.006 23911423

[24] Zea AH, Ochoa MT, Ghosh P, Longo DL, Alvord WG, Valderrama L et al Changes in Expression of Signal Transduction Proteins in T Lymphocytes of Patients with Leprosy. Infection and immunity. 1998; 66(2):499–504. 9453602 10.1128/iai.66.2.499-504.1998 PMC107934

[25] Pereira RMS, Calegari-Silva TC, Hernandez MO, Saliba AM, Redner P, Pessolani MCV et al Mycobacterium leprae induces NF-jB-dependent transcription repression in human Schwann cells. Biochemical and Biophysical Research Communications. 1995; 335: 20–26. 10.1016/j.bbrc.2005.07.061 16055086

[26] Adams MN, Ramachandran R, Yau MK, Suen JY, Fairlie DP et al Structure, function and pathophysiology of protease activated receptors. Pharmacol Ther. 2011; 130: 248–282. 10.1016/j.pharmthera.2011.01.003 . 21277892

[27] Aerts L, Hamelin M-È, Rhéaume C, Lavigne S, Couture C, et al Modulation of Protease Activated Receptor 1 Influences Human Metapneumovirus Disease Severity in a Mouse Model. PLoS ONE. 2013; 8(8): e72529 10.1371/journal.pone.0072529 24015257 PMC3755973

[28] Chionh YT, Ng GC, Ong L, Arulmuruganar A, Stent A, Saeed MA, Wee JLK, Sutton P. Protease-activated receptor 1 suppresses Helicobacter pylori gastritis via the inhibition of macrophage cytokine secretion and interferon regulatory factor 5. Mucosal Immunology. 2014 10.1038/mi.2014.43 24866378

[29] Arnaud E, Nicaud V, Poirier O. Protective effect of a thrombin receptor (protease-activated receptor 1) gene polymorphism toward venous thromboembolism. Arterioscler Thromb Vasc Biol. 2000; 20: 585–592 10669659 10.1161/01.atv.20.2.585

[30] Klingler K, Tchou-Wong KM, Brandli O. Effects of mycobacteria on regulation of apoptosis in mononuclear phagocytes. Infect Immun. 1997; 5:5272–5278. 10.1128/iai.65.12.5272-5278.1997 PMC175759 9393826

[31] Chattree V, Khanna N, Bisht V, Rao DN. Inhibition of apoptosis, activation of NKT cell and upregulation of CD40 and CD40L mediated by M. leprae antigen(s) combined with Murabutide and Trat peptide in leprosy patients. Mol Cell Biochem. 2008; 309:87–97. 10.1007/s11010-007-9646-8 18008143

[32] Sun T, Gao Y, Tan W, Ma S, Shi Y, et al A six-nucleotide insertiondeletion polymorphism in the CASP8 promoter is associated with susceptibility to multiple cancers. Nat Genet. 2007; 39: 605–613. 17450141 10.1038/ng2030

[33] Teles RMB, Krutzik SR, Ochoa MT, Oliveira RB, Sarno EN, Modlin RL. Interleukin-4 Regulates the Expression of CD209 and Subsequent Uptake of Mycobacterium leprae by Schwann Cells in Human Leprosy. Infection and immunity. 2010; 78(11):4634–4643. 10.1128/IAI.00454-10 20713631 PMC2976321

[34] Nakashima H, Miyake K, Inoue Y. Association between IL4 genotype and IL-4 production in the Japanese population. Genes Immun. 2002; 3:107–9. 11960309 10.1038/sj.gene.6363830

[35] Beitelshees AL, Johnson JA, Hames ML, Gong Y, Cooper-DeHoff RM, et al Aromatase Gene Polymorphisms Are Associated with Survival among Patients with Cardiovascular Disease in a Sex-Specific Manner. PLoS ONE. 2010 5(12): e15180 10.1371/journal.pone.0015180 21170323 PMC3000815

[36] Foss NT, Motta ACF. Leprosy, a neglected disease that causes a wide variety of clinical conditions in tropical countries. Mem Inst Oswaldo Cruz. 2012; 107: 28–33. 23283450 10.1590/s0074-02762012000900006

[37] Leal AMO, Magalhaes PKR, Souza CS, Foss NT. Adrenocortical hormones and interleukin patterns in leprosy. Parasite Immunol. 2003; 25: 457–461. 14651593 10.1111/j.1365-3024.2003.00654.x

[38] Limer KL, Pye SR, Thomson W, Boonen S, Borghs H, Vanderschueren D et al Genetic Variation in Sex Hormone Genes Influences Heel Ultrasound Parameters in Middle-Aged and Elderly Men: Results From the European Male Aging Study (EMAS). J. Bone Miner Res. 2009; 24(2): 314–323. 10.1359/jbmr.080912 18767927

[39] Eidt LM. Breve história da hanseníase: sua expansão do mundo para as Américas, o Brasil e o Rio Grande do Sul e sua trajetória na saúde pública brasileira. Saúde Soc. 2004; 13:76–88.

[40] Lastória JC, Abreu MAMM. Leprosy: review of the epidemiological, clinical, and etiopathogenic aspects—Part 1. An Bras Dermatol. 2014; 89(2):205–18. 10.1590/abd1806-4841.20142450 24770495 PMC4008049

[41] Scott HH. The influence of the slave-trade in the spread of tropical disease. Transactions of the society of tropical and hygiene medicine. Trans R Soc Trop Med Hyg. 1943; 37(3):169–188.

[42] Ribeiro-dos-Santos AK, Carvalho BM, Feio-dos-Santos AC, Santos SE. Nucleotide variability of HV-I in Afro-descendants populations of the Brazilian Amazon Region. Forensic Sci Int. 2007; 167:77–80. 16448796 10.1016/j.forsciint.2005.12.033

[43] Ribeiro-Rodrigues EM, dos Santos NP, dos Santos AK, Pereira R, Amorim A, Gusmão L, Zago MA, Santos SE. Assessing interethnic admixture using na X-linked insertion-deletion multiplex. Am J Hum Biol. 2009; 21:707–709. 10.1002/ajhb.20950 19533621

[44] Santos SEB, Rodrigues JD, Ribeiro-dos-Santos AK, Zago MA. Differential contribution of indigenous men and women to the formation of an urban population in the Amazon region as revealed by mtDNA and Y-DNA. Am J Phys Anthropol. 1999; 109:175–180. 10378456 10.1002/(SICI)1096-8644(199906)109:2<175::AID-AJPA3>3.0.CO;2-#

[45] Ribeiro-dos-Santos AKC, Carvalho BM, Santos ACF, Santos SEB. Nucleotide variability of HV-I in Afro-descendents populations of the Brazilian Amazon Region. Forensic Science International. 2007; 167: 77–80. 16448796 10.1016/j.forsciint.2005.12.033

[46] Han XY, Silva FJ. On the Age of Leprosy. PLoS Negl Trop Dis. 2014; 8(2): e2544 10.1371/journal.pntd.0002544 24551248 PMC3923669

