# Supplementary material for: Influence of Genetic Ancestry on INDEL Markers of NFKβ1, CASP8, PAR1, IL4 and CYP19A1 Genes in Leprosy Patients
Source: PLoS Negl Trop Dis. 2015 Sep 14;9(9):e0004050. doi: 10.1371/journal.pntd.0004050 (PMC4569399; doi:10.1371/journal.pntd.0004050)
Supplement: S1 Table — (DOCX) [file pntd.0004050.s001.docx]

**S1 Table**. Ranges of African and European genetic ancestry contributions in leprosy patients and healthy individuals.

| **African** | **Leprosy patients (N=141)** | | **Healthy Individuals (N=180)** | |  |  |
| --- | --- | --- | --- | --- | --- | --- |
| **Range (%) of contribution** | Percent | Cumulative Percent | Percent | Cumulative Percent | *p value***^a^** | OR (95% CI)^b^ |
| 0 ├─ 10 | 4.3 | 4.3 | 1.1 | 1.1 |  | 1 |
| 10├─ 20 | 24.1 | 28.4 | 41.7 | 42.8 | 0.035 | 0.93 (1.00-0.76) |
| 20├─ 30 | 37.6 | 66 | 40 | 82.8 | 0.010 | 0.41 (0.85-0.15) |
| 30├─ 40 | 17 | 83 | 12.2 | 95 | 0.001 | 0.38 (0.75-0.10) |
| 40├─ 50 | 9.9 | 92.9 | 4.4 | 99.4 | 0.005 | 0.17 (0.82-0.12) |
| 50├─ 60 | 5.7 | 98.6 | 0.6 | 100 | - | - |
| 60├─ 70 | 1.4 | 100 | - | - | - | - |

| **European** | **Leprosy patients (N=141)** | | **Healthy Individuals (N=180)** | |  |  |
| --- | --- | --- | --- | --- | --- | --- |
| **Range (%) of contribution** | Percent | Cumulative Percent | Percent | Cumulative Percent | *p value***^a^** | OR (95% CI)^b^ |
| 0 ├─ 10 | 1.4 | 1.4 | 1.1 | 1.1 |  | 1 |
| 10├─ 20 | 2.8 | 4.3 | 5 | 6.1 | 0.001 | 1.25 (1.01-1.86) |
| 20├─ 30 | 12.1 | 16.3 | 16.1 | 22.2 | 0.009 | 1.63 (1.05-1.98) |
| 30├─ 40 | 22 | 38.3 | 33.3 | 55.6 | 0.035 | 1.80 (1.25-2.10) |
| 40├─ 50 | 27.7 | 66 | 31.7 | 87.2 | 0.026 | 2.22 (1.85-3.5) |
| 50├─ 60 | 17 | 83 | 12.8 | 100 | 0.045 | 2.45 (1.23-12.5) |
| 60├─ 70 | 12.1 | 95.1 | - | - | - | - |
| 70├─ 80 | 5 | 100 | - | - | - | - |

**^a^***p value* obtained for logistic regression; ^b^odds ratio (OR).
